# Clinical Biofilm Ring Test^®^ Reveals the Potential Role of β-Lactams in the Induction of Biofilm Formation by *P. aeruginosa* in Cystic Fibrosis Patients

**DOI:** 10.3390/pathogens9121065

**Published:** 2020-12-19

**Authors:** Elodie Olivares, Jason Tasse, Stéphanie Badel-Berchoux, Christian Provot, Gilles Prévost, Thierry Bernardi

**Affiliations:** 1 Virulence Bactérienne Précoce UR7290, ITI Innovec, FMTS, Institut de Bactériologie, Université de Strasbourg, 3 rue Koeberlé, 67000 Strasbourg, France; prevost@unistra.fr; 2 BioFilm Pharma SAS, 317 Avenue Jean Jaurès, 69007 Lyon, France; jason.tasse@biofilmpharma.com (J.T.); christian.provot@gmail.com (C.P.); thierry.bernardi@biofilmpharma.com (T.B.); 3 BioFilm Control SAS, Rue Emile Duclaux, 63360 Saint Beauzire, France; stephanie.badel@biofilmcontrol.com

**Keywords:** biofilm, cystic fibrosis, *Pseudomonas aeruginosa*, antibiotics, clinical Biofilm Ring Test^®^, early bacterial adhesion induction

## Abstract

Biofilms are characterized by high tolerance to antimicrobials. However, conventional antibiograms are performed on planktonic microorganisms. Through the clinical Biofilm Ring Test^®^ (cBRT), initially aimed to measure the adhesion propensity of bacteria, we discerned a variable distribution of biofilm-producer strains among *P. aeruginosa* samples isolated from expectorations of cystic fibrosis (CF) patients. Despite a majority of spontaneous adherent isolates, few strains remained planktonic after 5 h of incubation. Their analysis by an adapted protocol of the cBRT revealed an induction of the biofilm early formation by sub-inhibitory doses of β-lactams. Microscopic observations of bacterial cultures stained with Syto 9/Propidium Iodide (PI) confirmed the ability of antimicrobials to increase either the bacterial biomass or the biovolume occupied by induced sessile cells. Finally, the cBRT and its derivatives enabled to highlight in a few hours the potential inducer property of antibiotics on bacterial adhesion. This phenomenon should be considered carefully in the context of CF since patients are constantly under fluctuating antimicrobial treatments. To conclude, assays derived from the Biofilm Ring Test^®^ (BRT) device, not only define efficient doses preventing biofilm formation, but could be useful for the antimicrobial selection in CF, to avoid inducer molecules of the early biofilm initiation.

## 1. Introduction

*Pseudomonas aeruginosa* is highly resistant to antimicrobials and plays a major role in nosocomial infections leading to a high mortality rate [1,2]. Its survival ability is very complex and involves multifactorial mechanisms including the capacity to grow in biofilms. These structural organizations are defined as communities of microorganisms embedded in a self-produced matrix and adhering to surfaces. Biofilm-growing *P. aeruginosa* is frequently found in cystic fibrosis (CF) patients where it causes severe lung damages and difficult-to-treat chronic infections [3]. Indeed, it is now well recognized that Minimal Inhibitory Concentrations (MICs) and Minimal Bactericidal Concentrations (MBCs) of antimicrobials effective against bacteria in biofilms may be 10 to 1000-fold higher than those effective against the planktonic microorganisms [4,5]. This tolerance can be explained, at least partly, by the slower metabolism of sessile bacteria forming the biofilm, the hypoxic environment inside these structures, and the presence of a persistent subpopulation of microbes [6]. Previous works also demonstrated that antimicrobials act as signal agents [7,8]. At low concentrations, antimicrobials can be beneficial to bacterial survival in nature by increasing the expression of virulence determinants. Then, they act as signal molecules for the homeostasis regulation of microbial communities. In this way, numerous recent studies demonstrated that antimicrobial subinhibitory concentrations trigger the expression of factors contributing to the biofilm formation by sessile bacteria [9,10]. The consequence in CF patients, as they are usually under variable concentrations of antimicrobials, could be the selection of multi-drug resistant *P. aeruginosa* strains associated or not with the induction of biofilm formation, thereby favoring the move towards chronicity of the disease [11]. This premise means that the optimization of anti-*Pseudomonas* chemotherapy should be considered.

In a previous study, we evaluated the biofilm formation ability of *P. aeruginosa* isolates that originated from the sputum of anonymized CF patients, by using the BioFilm Ring Test (BRT) technology (BioFilm Control, Saint-Beauzire, France) [12]. Among the ones which spontaneously settle in biofilms, it also highlighted a prevention process of the early bacterial adhesion by amikacin and tobramycin [13]. In the present work, we similarly used an alternative protocol of the BRT, the clinical Biofilm Ring Test (cBRT), on our clinical *P. aeruginosa* strains. This assay, based on the paramagnetic microbead migration in the presence or absence of adherent bacteria, was initially devised for the profiling of biofilm-producing strains through the analysis of their serial diluted cultures [14]. Among strains classified as non-adherent, we documented an induction process of the early steps of biofilm production by β-lactam sub-MICs. Epifluorescence microscopy, allowing direct observation of microorganisms growing with low doses of antimicrobials, confirmed that antibiotics could either induce the early adhesion of bacterial cells or promote their switch into a probably more virulent filamentous form.

## 2. Results

### 2.1. Microbial Biofilm Profiling with cBRT

**Figure 1:**
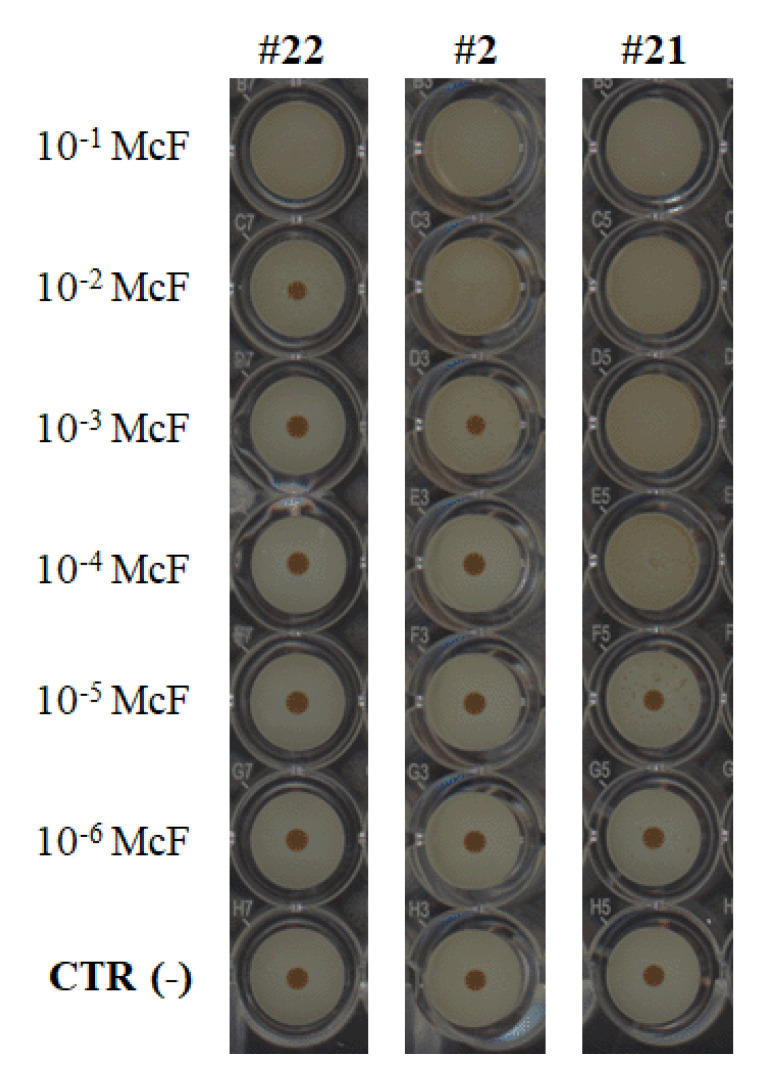
Biofilm profiling of *P. aeruginosa* clinical isolates according to their ability to block the migration of magnetic microbeads in clinical Biofilm Ring Test (cBRT) assay. Biofilm formation in 96-well polystyrene plates was shown after 5 h of incubation at 37 °C for representative strains of three adhesion profiles: strain #22 (no-biofilm producer), strain #2 (weak-biofilm producer) and strain #21 (moderate-biofilm producer). A gradual blocking of beads was noticed depending on the microbial serial dilutions and therefore reflecting the strength of the biofilm produced by bacteria. Images were obtained after magnetization of the plates on the block test and scanning with the plate reader. Negative controls with only Brain Heart Infusion (BHI) medium and magnetic microparticles were displayed in the last line of microplates.

Initially, the cBRT procedure allowed for the characterization of microbial cultures through the biofilm strength evaluation of the bacterial serial dilutions. If a low-bacterial-content suspension is not sufficiently strong to entrap the magnetic beads moving under the magnetic field forces, this indirectly reflects the weak ability of the strain to form a biofilm. Conversely, if at a low concentration the bacterial cells can efficiently block the microbeads, it defines a strong biofilm producer. Examples of diversified behaviors of bacteria are suggested in Figure 1. The three isolates showed a gradual blocking of the microbeads according to the culture dilutions, which reflected the strength of the biofilm formed.

**Figure 2:**
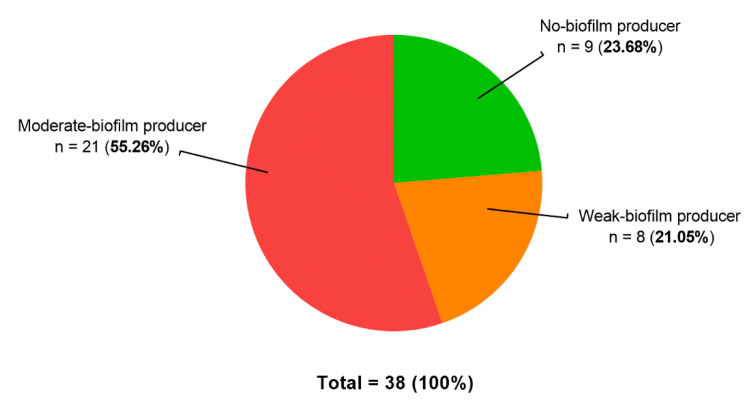
Distribution of adhesion profiles among 38 clinical strains of *P. aeruginosa*. The cBRT assay allows for the categorization of bacteria according to their biofilm strength. Through the calculation of Biofilm-forming Potential (BP) values, clinical isolates were classified as no-, weak-, moderate- or high-biofilm producer. No high-biofilm producer strains were found in this panel.

The 38 clinical strains of *P. aeruginosa* included in the study were split into three distinct adhesion profiles (Figure 2). Globally, bacterial isolates are enabled to spontaneously initiate the formation of a biofilm as 21.05% and 55.26% of them were classified as weak- and moderate-biofilm producers, respectively. This revealed that more than 75% of *P. aeruginosa* samples were naturally sessile. This proportion confirmed its inherent ability to adhere to surfaces, particularly in the CF context. Among our collection, none of the strains were found to be able to block the bead migration at the lowest bacterial dilutions, corresponding to the high-biofilm producer category. Interestingly, nine isolates appeared to remain in a planktonic form after 5 h of incubation (23.68%). We chose to further document the potential impact of β-lactam antibiotics with four non-adherent strains (#6, #20, #22 and #31). They were selected as they showed a stable planktonic phenotype, allowing for a highly reproductible analysis of the biofilm induction by antimicrobials (strain-dependent process).

### 2.2. Early Bacterial Adhesion Induction by Antimicrobials Evidenced with Modified cBRT Assay

Compared to the previous cBRT procedure, allowing the discrimination between biofilm-producing strains depending on their adhesion strength, the adapted protocol specifies the impact of antimicrobials on the inherent adhesion phenotype of bacteria. After 5 h of incubation, in the absence of antibiotics, three of the four tested *P. aeruginosa* strains (#6, #22, and #31) were able to partially immobilize the magnetic microbeads only at the highest cell concentration (10^−1^ McF), suggesting, as above, a low spontaneous biofilm formation ability from the bacteria (Table 1A,C,D). The Biofilm-forming Potential (BP) value of the strain #20 positive control, averaged from three experiments with antimicrobials, categorized the clinical isolate as a weak-biofilm producer (Table 1B).

The analysis of diluted microbial cultures complemented with low doses of antibiotics allowed for evaluating the effect of antimicrobials even if bacteria were spontaneously poorly adherent. For the four tested remaining-planktonic strains, an induction phenomenon was observed for at least one antibiotic molecule at 0.1 × MIC. Usually, it led to one category discrepancy compared to the initial one, which defined the inherent behavior of bacteria. Only imipenem favored the adhesion of strain #31 (Table 1D). For strain #22, this carbapenem was ineffective, while all other molecules (ticarcillin-clavulanate (TIM), cefepime (FEP), ceftazidime (CAZ), and meropenem (MEM)) modified the biofilm potential of bacteria, as they evolved from the class of non- to weak-biofilm producer (Table 1C). Strain #20 was also stimulated for biofilm production by TIM, FEP, CAZ, and imipenem (IPM) (passage from the weak- to the moderate-biofilm producer category) but not by MEM, which can be interpreted by an absence of induction, or an induction level not detectable by the 10-fold dilutions of inocula (Table 1B). Finally, a substantial induction process was obtained with planktonic strain #6. It was found to adhere more readily to the well bottoms at McF = 10^−2^ when TIM, FEP, and CAZ were added to cultures before starting incubation, identifying this strain as a new weak-biofilm producer. However, the more dramatic classification change was observed with both carbapenems. Isolated bacteria, which were initially not able to completely block the magnetic beads at 10^−1^ McF, could immobilize them at a more diluted concentration of 10^−3^ McF when IPM and MEM were present at 0.1× MIC in the microbial suspensions (Table 1A).

These category variations were significant regarding the notion of the biofilm formation induction since each modification relied on the difference of at least one log of the bacterial concentration for the tested condition. To further explore mechanisms responsible for the spot absence in wells with low antimicrobial concentrations, reflecting a potential induction of biofilm production, microscopic experiments were conducted.

### 2.3. Morphological Change and Increase in Covering Surface by Sessile Cells Corroborated in Epifluorescence Microscopy

**Figure 3:**
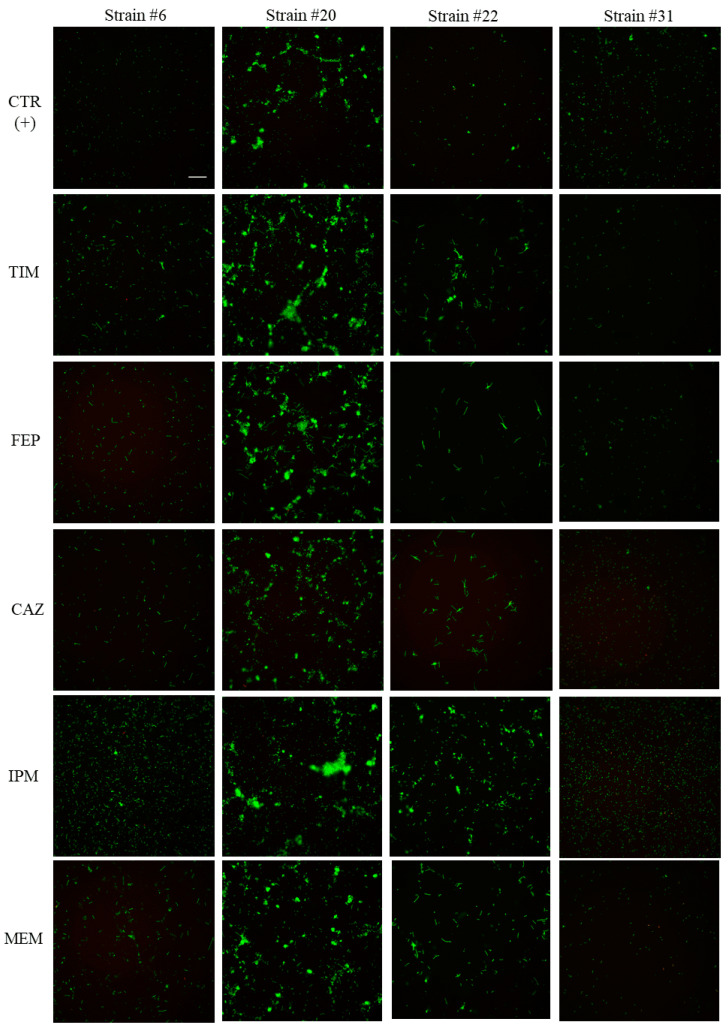
Representative images of adherent *P. aeruginosa* cells after 5 h of incubation, in the absence or presence of β-lactam molecules. Spontaneous adhesion of the clinical strains #6, #20, #22, and #31 was assessed by epifluorescence microscopy. Green fluorescence resulted from the staining of viable cells with Syto9 dye. Propidium iodide was also used to stain in red bacteria with compromised membranes but no dead cell was detected. Pictures are an overlay of both fluorescence channels. The addition of 0.1× Minimal Inhibitory Concentration (MIC) of antimicrobials to cultures could lead to either increased biomass of adherent bacteria or an alteration of the cell shape. Scale bar, 3 µm.

To verify if the adhesion induction by antimicrobials, previously observed with the cBRT assay, directly affects the *P. aeruginosa* growth and/or originates from a modification of the intrinsic behavior of bacteria, microscopic experiments of adherent cells stained with fluorescent dyes were performed. Representative images of bacterial cultures, supplemented with the sub-MICs of β-lactams, are provided in Figure 3. Fluorescent microscopy confirmed the previous classification of the clinical strains without antibiotics, i.e., that strains #6, #22, and #31 belong to the low-biofilm producer category. Indeed, very few bacilli were stained in the well bottoms after 5 h of incubation. Similarly, the slight spontaneous ability of strain #20 to block the magnetic microbead migration in cBRT visually manifested as the formation of green micro-colonies.

Examination of cultures with β-lactams revealed two distinct phenomena: either a modification of the cell shape or the stimulation of the bacterial adhesion. In fact, it could be easily noticed that bacteria of strains #6 and #22 appeared elongated when they were in the presence of TIM, FEP, CAZ, and MEM, compared to the normal cell morphology of the control suspensions. However, this size elongation seemed to be a strain-dependent process as it was not noticed with strain #31 regardless of antimicrobials. To validate these visual observations, microscopy pictures were analyzed according to the ImageJ software. By image examination, the pixel number depicting a fluorescent object can be converted to a value in µm and the number of bacterial cells can be estimated. The average length and number of microbial components present for the control strains and cultures with β-lactams are summarized in Table 2.

Compared to the normal size of cells documented for strain #6 (0.9 µm on average), a statistically significant increase in bacteria length, ranging from 175% to nearly 250%, was noticed for cultures with TIM, FEP, CAZ, and MEM. This reflected a filamentous form of induction by antimicrobials. The cell elongation of strain #22 was significantly more substantial as the bacteria in antibiotic conditions were at least 300% longer than the control standard-sized cells (1.2 µm). In contrast, the IPM molecule did not seem to significantly affect the bacteria size, compared to the other antimicrobials. The microscopic observation of wells for this antibiotic rather showed the presence of many more sessile cells. While few bacilli were able to spontaneously adhere, this carbapenem led to a statistically significant increase in the surface covered by bacterial biomasses of strains #6, #22 and #31. The fluorescent objects could easily be quantified by the image processing software and the data correlated with these observations. For strains #6, #22, and #31, the adherent cells grown with IPM were at least twice as numerous than the corresponding control cell layers. As strain #20 was spontaneously able to agglomerate, the fluorescent analysis was not focused on individual cells but was adjusted on the stained clusters. The preliminary look of pictures supposed that bacteria with β-lactam antimicrobials gathered in structures whose global shape was higher than the one of the control clusters (except for CAZ) but their corresponding statistical analysis did not conclude to a significant size difference. This correlation absence between eye observation and statistical data could be because the calculations were made on averaged sizes of bacterial clusters while the formation of bacterial micro-colonies, whose dimensions were random, was not a standardized process. Consistent with the use of sub-MICs, no dead cells were seen with Propidium Iodide (PI) staining.

## 3. Discussion

In the hospital routine, the analysis of bacteria antibiotic susceptibility is performed on planktonic cells by the MIC determination of antimicrobials. From these concentrations, international committees such as CASFM/EUCAST in France, EUCAST for Europe, or CLSI for the USA offer clinical breakpoint tables, allowing the strain classification to be divided into susceptibility testing categories: S (susceptible), I (intermediate redefined as susceptible at increased exposure since 2019) and R (resistant) [15]. In this study, the four clinical isolates of *P. aeruginosa* were considered as belonging to the I- or R-group for almost all tested antibiotics. At first glance, a correlation between the antimicrobial susceptibility profile of bacterial strains in a planktonic form and the behavior of their biofilms to antibiotic molecules did not exist. The biofilm stimulation, as an adaptative response of bacteria to antibiotics, was even described for strains initially susceptible to these same molecules [16,17]. This required independently considering the spontaneous growth of microorganisms in biofilms, especially as their antibiotic tolerance is much higher than the one of isolated cells. The development of technologies dedicated to the evaluation of antimicrobial efficacy on adherent bacteria becomes crucial, especially for the optimization of anti-*Pseudomonas* chemotherapies in CF [18].

In the present work, we initially sought to document the adhesion profile of a collection of CF strains of *P. aeruginosa* with the cBRT, which enabled the rapid profiling of microbial isolates through the biofilm strength [14]. Preliminary results evidenced the spontaneous ability of most of the bacteria to rapidly initiate the formation of a strong biofilm as only a few strains remained non-adherent after 5 h of incubation. Previous studies had already described this inherent ability of *P. aeruginosa* clinical isolates to form biofilms by standard Crystal Violet (CV) assay. This tissue culture plate method initially assessed the quantification of biofilm by the bacteria categorization according to their adhesion strength, as for the cBRT device. Eladawy et al. evaluated the potential preventive effect of lysozyme and proteinase K on the biofilm of a collection of 103 *P. aeruginosa* clinical isolates [19]. Among them, an analysis of 13 strains originated from sputum through CV assay revealed that 10 were even strongly-adherent, 3 moderately-adherent, and 1 weakly-adherent. Although the isolate origin was mentioned, the authors did not specify whether sputum arose from CF patients. In an anterior publication, the biofilm intensity of 35 *P. aeruginosa* isolates recovered from CF patients vs. 39 strains isolated from non-CF patients was also assessed with the CV method [20]. As for our collection, the authors found that 71% of samples were considered as being spontaneously adherent. Indeed, the distribution of weak- and moderate-biofilm producer profiles in our strains depicted 76% of them. Moreover, Perez et al. showed that these abilities to adhere and to produce biofilm in vitro were similarly shared by the non-CF isolates and were not specific to the CF pathology. The adhesion feature would be characteristic to all *P. aeruginosa* isolates recovered from sputa.

The inducer effect of β-lactams on the bacterial adhesion of four of these planktonic isolates was further assessed with the adapted cBRT procedure. In a clinical context, if the bacterial sample expresses a planktonic phenotype, an antibiotic inhibitory concentration of the biofilm formation cannot be defined but at-risk molecules may be identified. Mounting evidence suggesting that subinhibitory concentrations of antibiotics can stimulate the biofilm formation and affect pathogen resilience was recently reviewed [21]. The deleterious boost of the biofilm production is a strain- and antibiotic/concentration-dependent process, which can occur for CF patients when bacterial infections are treated with antimicrobials. In this specific pulmonary context, patients are prematurely and chronically colonized by *P. aeruginosa* and then are constantly under antibiotic treatments.

This negative stimulation of bacteria by antimicrobials probably does not originate from a gene mutation as for genetic variations causing resistance acquisition by bacteria [22,23]. It is likely a transient induction of gene expression, even if an epigenetic mechanism cannot be ruled out. Additional tests, performed with higher concentrations of a wider range of β-lactam molecules, revealed that the induction process is reversible (data not shown). Analyses of adhesion kinetics of stimulated bacteria showed that, in the few hours following the initial contact with antibiotics, bacteria restored their original adhesion behavior if antimicrobials were removed from the medium. It seems that the acquired sessile phenotype is not conserved by bacteria when antibiotic molecules are no longer present in cultures.

Two main changes were microscopically noticed in cell attitude when subinhibitory concentrations of β-lactams were added to initial cultures. Especially with the IPM molecule, we observed an increase in the sessile biomasses on the well bottoms with the formation of adherent mono-layers of bacteria. This was already noticed by Nucleo et al. with the *A. baumannii* clone exposed to IPM concentrations ranging from 1/16 to 1/4 of the MIC whose cell adhesion was stimulated by up to three-fold compared to the control culture [17]. A regular-morphological modification was also noted in microbial cultures with antibiotics. Indeed, the bacterial shape could be more elongated than one of the control strains. This induction of a filamentous form of bacteria by β-lactam antibiotics is an already-documented process [24]. Briefly, the primary target of these molecules is Penicillin-Binding Proteins (PBPs) in Gram-negative bacteria. These proteins initially catalyze the terminal stages in the assembly of the peptidoglycan network of the bacterial cell wall. Their inhibition could lead to the modification of cell shape into a spherical form or long filaments. Meropenem was described as having a high affinity for PBP-2 and PBP-3, resulting in the formation of filaments with or without an oval “bulge” at the center [25]. Owing to their high selective affinity for PBP-3, ceftazidime, and cefotaxime can also lead to the formation of long filaments in *Escherichia. coli*, *Klebsiella spp.*, *P. aeruginosa,* and *Acinetobacter spp.* [26]. Additionally, the assessment of BP-binding affinities for several cephalosporins revealed the role of cefepime in the cellular morphology of *E. coli*, by the induction of long filament formation at concentrations close to the MICs [27]. Finally, as we found that ticarcillin-clavulanate led to the cell elongation, Elliott et al. observed a similar morphological response of *P. aeruginosa* to ticarcillin and other β-lactam compounds [28]. Filamentation would also be combined with the transition of swimming cells to swarming motility. Indeed, swarming bacteria were described as becoming elongated and hyperflagellated [29,30]. Interestingly, this form of surface-associated translocation is involved in the early steps of biofilm establishment [31]. Bacterial strains capable of swarming movement form uniform biofilms while the others establish non-confluent layers. In a study defining *A. baumannii* clinical isolates on their motility features and biofilm production ability, the authors concluded that the two highest motile strains displayed a swarming-like translocation correlated with the more significant biofilm amounts [32].

Presently, the consecutive influence of sub-lethal doses of antibiotics on the antimicrobial resistance and tolerance of biofilms was documented in the literature [10,33]. Microorganisms, detecting a low antibiotic dose, could interpret it as a signal anticipating a future exposure to a higher concentration [34,35]. Antimicrobials seem to act particularly on the intracellular c-di-GMP levels and expression of genes involved in alginate metabolism, which can alter the biofilm formation ability of bacteria [36]. In the case of CF, Elliott et al. have also shown that approximately half of *P. aeruginosa* strains isolated from patients exhibited biofilm induction phenomena [37]. In vivo, these effects could be common, as bacterial strains are exposed to low antibiotic concentrations at the very beginning and end of standard treatment, between doses, or continuously during low-dose therapy [38]. Nonetheless, the cBRT and more generally the BRT-derived assays appear to be efficient to easily highlight this unsafe phenomenon in a few hours, by the detection of the early adhesion of bacteria in a clinical sample. As a whole, in a clinical context, these procedures could discriminate between the risk-free antimicrobials and the molecules inducing the biofilm production, which would be precious data for the clinicians.

## 4. Conclusions

Compared to available conventional methods, which do not allow this information to be recovered so rapidly, standardized use of the BRT device in hospital labs seems to be highly promising. To closely reflect the spontaneous biofilm mode of *P. aeruginosa* within the CF lung, antibiotic regimens based on the sessile cell behavior must be considered, as the selection of treatments should be different according to the susceptibility test used. Simulated regimens based on biofilm or conventional planktonic methods can yield contradictory prescriptions [39]. In this sense, BRT assays appear to be complementary to antibiograms performed in routine hospital practice by adding a biofilm risk assessment associated with antibiotics. The clinical significance of all these results remains to be specified. Additional studies on antibiotic-induced biofilm formation might elucidate involved mechanisms and dose-dependent behaviors. More selection and genomic investigations are also needed to understand mechanisms sustaining categories of biofilm inducers, according to antimicrobials.

## 5. Materials and Methods

### 5.1. Bacterial Strains

We used a collection of 38 clinical *P. aeruginosa* strains isolated from the sputum of CF anonymized patients to perform standard cBRT. Antibiotic susceptibility testing with a modified application of the cBRT assay and microscopy experiments were performed on 4 non-adherent isolates (#6, #20, #22, and #31). The induction of the early steps of biofilm formation by antimicrobials being a strain-dependent process, these strains were selected as they showed significant data with in vitro assays. The whole *P. aeruginosa* strains originated from the sputum of CF anonymized patients and culture stocks were conserved in glycerol at −80 °C. They were subcultured on Brain Heart Infusion (BHI) agar plates before the realization of the different assays.

### 5.2. Antibiotic Selection and Minimum Inhibitory Concentration (MIC) Determination

The induction of the early steps of biofilm formation by β-lactams was investigated with the modified cBRT assay (and confirmed with microscopy) using 0.1 Minimal Inhibitory Concentrations (MICs) of antibiotics. The molecule panel was composed of ticarcillin-clavulanate (TIM), cefepime (FEP), ceftazidime (CAZ), imipenem (IPM), and meropenem (MEM) (EDQM, France). The initial MICs of antimicrobials were previously determined on the planktonic strains #6, #20, #22, and #31 by gradient diffusion strips, following supplier’s indications (bioMérieux, Marcy-l’Étoile, France and Liofilchem, Waltham, MA, USA). They are summarized in Table 3.

### 5.3. Clinical Biofilm Ring Test (cBRT)

The cBRT protocol was developed to concomitantly characterize several biofilm-producing microbial strains [14]. Its principle is based on the aggregation measurement of magnetic microbeads in wells of a 96-well microplate, under a magnetic field generated by mini-magnets placed under each well bottom of the plate. The total or partial reduction of the bead aggregation reflects the microbial biofilm formation initiation. Indeed, the distinctive feature of the cBRT tool consists of serial 10-fold dilutions of inoculum to assess bacteria propensity to adhere by comparing beads blocking in weaker and weaker inocula conditions. The device kit consists of 96-well microplates, TON004 (toner suspension containing magnetic microbeads), contrast liquid LIC001 (inert opaque oil used for plate reading step), and a pack composed of a block test (magnet holder), a dedicated plate reader (BioFilm Reader) and the BioFilm Control Elements^®^ 3.0 software to acquire and analyze images.

Fresh overnight cultures of clinical isolates were used to inoculate 3 mL of sterile ultra-pure water to the equivalent of 1.0 McFarland (McF) turbidity standard. A total of 100 µL/well of bacterial suspensions were dropped in the first line of a 96-well microplate. Each strain was tested in column duplicate. In the remainder of the plate, a mixed solution of BHI and Toner suspension (10 µL/mL) were added in dedicated columns in a volume of 200 µL per well.

Sets of 10-fold serial dilutions, from 1.10^−1^ to 1.10^−6^ McF, were done; the 1 McF-initial culture being considered as the “pure” suspension. The last line of the microplate was kept for the negative control (only BHI with beads) to check the absence of contamination and to participate in the data interpretation by calculating the Biofilm-forming Potential (BP) for each tested condition.

The microplates were incubated at 37 °C for 5 h and the assays performed in duplicates. For the plate reading, the wells were covered by 120 µL of the contrast liquid solution. Then, the microplate was magnetized for one minute with the block test and scanned with the BioFilm Reader. The magnetic field enables free beads attraction towards the center of the well, forming a brown spot made of aggregated particles, while beads embedded in a biofilm are blocked and remain non-detectable. The adhesion ability of bacteria was expressed as the Biofilm Formation Index (BFI) calculated by the BioFilm Control Elements^®^ 3.0 software. The Biofilm-forming Potential (BP), namely, the ability of bacteria to form biofilm ranging from 0 to 1, was specifically developed by Di Domenico et al. for the cBRT assay and is determined as BP = [1 − (BFI sample/average BFIs of negative control)] [14]. Tested condition(s) being analyzed in duplicate, the average BP was calculated for each bacterial concentration and compared to the specific cut-off (BPc) value of the assay. The latter is equal to 1 − [((average BFIs of negative control − three standard deviation)/2)/average BFIs of negative control]. It assumes that if a strain, in a specific cell concentration, has a BP which is approximately more than a half of that measured for the negative control, it can be considered a biofilm producer. The global ability of the strain to form biofilm is identified by its first concentration for which the BP value is higher than the cut-off. Thereby, it leads to its categorization into the following categories: BP < BPc at 10^−1^ McF = no-biofilm producer; BP > BPc at 10^−1^ and/or 10^−2^ McF = weak-biofilm producer; BP > BPc at 10^−3^ and/or 10^−4^ McF = moderate-biofilm producer and BP > 10^−5^ and/or 10^−6^ McF = high-biofilm producer.

### 5.4. cBRT with Antibiotics

An adaptation of the cBRT protocol described above was subsequently carried out for the behavior characterization of non-adherent *P. aeruginosa* strains in the presence of 0.1 × MIC of β-lactam molecules, aiming to assess the impact of antibiotics on cell adhesion. It was derived from the previous one and was performed using the same reagents. The overall procedure was equivalent except for the preparation of the mix solution of BHI, which was directly complemented with subinhibitory doses of β-lactams (see Table 3 for the specific concentrations) in addition to Toner suspension (10 µL/mL). Bacterial cultures were incubated 5 h at 37 °C without shaking and the microplate was analyzed according to the manufacturer’s recommendations.

### 5.5. Staining of Adherent Bacteria and Fluorescence Microscopic Image Acquisition

Epifluorescence microscopy associated with staining of adherent microorganisms enabled the direct visualization and consequent quantification of sessile cells after antibiotic treatment.

Sterile untreated 6-well plates (Costar^®^, Washington, DC, USA) were inoculated with initial bacterial suspensions prepared from fresh BHI-agar cultures. Culture medium (2 mL adjusted to 0.3 McF) was used to inoculate the plate wells, supplemented or not with 0.1xMIC of antimicrobial molecules. These inoculi should be more concentrated than the microbial conditions for which an induction process was observed in cBRT assay, as the microscopy method, which required several washing steps, could lead to a removal of sessile cells.

The culture plates were maintained at 37 °C for 5 h. After incubation, supernatants were removed and remaining adherent cells were stained with Syto9 (ThermoFischer Scientific, Illkirch-Graffenstaden, France) combined with Propidium Iodide (PI) (Sigma-Aldrich Chimie S.a.r.l, Saint-Quentin-Fallavier, France). Both stains were added to culture plate wells at a dilution of 0.1 µL/mL for Syto9 and 1 µL/mL for PI, both in sterile ultra-pure water. The staining was performed protected from direct light for 15 min. Wells were washed with phosphate-buffered saline (1×, pH 7.2) and bacterial mono-layers were fixed with 4% formaldehyde for 10 min in darkness. Well bottoms could then be observed using an epifluorescence microscope (Olympus System Microscope Model BX60 with immersion objective ×40 N.A. 0,80). Acquisition of pictures was performed with an Orcaflash 4.0 camera (C11440, Hamamatsu, Massy, France) and HC Image Live software (Hamamatsu). At least three microscopic fields were acquired for each well. The corresponding pictures were analyzed according to the free software ImageJ (NIH), which allowed for the viewing and the discrimination of live vs. dead bacteria. For one microscopic field, two images were taken with two couples of excitation/emission filters: the GFP-FITC one (470–510 nm) for the revelation of Syto9 green fluorescence and the Texas Red one (585–624 nm) for the detection of red bacteria stained with PI. The software also allowed us to numerically quantify microorganisms/clusters present per microscopic field through the calculation of pixel number. The corresponding variation rates (in %) were also specified to estimate a change in the cell length or in the bacterial body number of microbial cultures with antibiotics, compared to the values found with control conditions. They were calculated with the formula (antibiotic value − control value)/control value × 100. Experiments were performed in triplicates. One-way ANOVA analyses and Dunnett’s multiple comparison tests were applied to statistically compared data of antibiotic conditions with the control ones for each clinical isolate.

## Figures and Tables

**Table 1:** Overall results of adhesion categorization for (**A**) strain #6, (**B**) strain #20, (**C**) strain #22, and (**D**) strain #31 obtained with the cBRT assay.

**(A) McF**	**CTR (+)**	**TIM**	**FEP**	**CAZ**	**IPM**	**MEM**	
10^−1^	**0.71 (*0.26*)**	1.00 (*0.00*)	0.81 (*0.17*)	0.86 (*0.12*)	1.00 (*0.00*)	1.00 (*0.00*)	**No**
10^−2^	0.29 (*0.24*)	**0.94 (*0.06*)**	**0.76 (*0.23*)**	**0.71 (*0.18*)**	1.00 (*0.00*)	1.00 (*0.00*)	**Weak**
10^−3^	−0.03 (*0.08*)	0.52 (*0.16*)	0.48 (*0.26*)	0.50 (*0.14*)	**0.75 (*0.08*)**	**0.77 (*0.05*)**	**Moderate**
10^−4^	−0.10 (*0.13*)	0.27 (*0.21*)	0.21 (*0.20*)	0.24 (*0.17*)	0.13 (*0.12*)	0.13 (*0.14*)	Moderate
10^−5^	−0.08 (*0.13*)	0.10 (*0.17*)	0.23 (*0.18*)	0.24 (*0.18*)	0.07 (*0.06*)	0.04 (*0.01*)	High
10^−6^	−0.09 (*0.14*)	0.03 (*0.23*)	0.18 (*0.15*)	0.17 (*0.13*)	0.02 (*0.09*)	−0.08 (*0.09*)	High
Average **BPc = 0.71 (*0.12*)**.							
**(B) McF**	**CTR (+)**	**TIM**	**FEP**	**CAZ**	**IPM**	**MEM**	
10^−1^	1.00 (*0.00*)	1.00 (*0.00*)	1.00 (*0.00*)	1.00 (*0.00*)	1.00 (*0.00*)	1.00 (*0.00*)	No
10^−2^	**1.00 (*0.00*)**	1.00 (*0.00*)	1.00 (*0.00*)	1.00 (*0.00*)	1.00 (*0.00*)	1.00 (*0.01*)	Weak
10^−3^	0.50 (*0.29*)	**0.67 (*0.12*)**	**0.69 (*0.23*)**	**0.70 (*0.15*)**	**0.86 (*0.19*)**	0.52 (*0.18*)	**Moderate**
10^−4^	0.00 (*0.02*)	0.01 (*0.06*)	0.02 (*0.04*)	0.02 (*0.02*)	0.18 (*0.29*)	0.03 (*0.07*)	Moderate
10^−5^	0.00 (*0.02*)	0.01 (*0.03*)	0.02 (*0.05*)	0.01 (*0.02*)	0.01 (*0.05*)	0.04 (*0.08*)	High
10^−6^	0.00 (*0.02*)	0.03 (*0.04*)	0.04 (*0.07*)	0.03 (*0.02*)	0.04 (*0.09*)	−0.01 (*0.02*)	High
Average **BPc = 0.57 (*0.02*)**							
**(C) McF**	**CTR (+)**	**TIM**	**FEP**	**CAZ**	**IPM**	**MEM**	
10^−1^	**0.98 (*0.03*)**	1.00 (*0.00*)	1.00 (*0.00*)	1.00 (*0.00*)	**1.00 (*0.00*)**	1.00 (*0.00*)	**No**
10^−2^	0.22 (*0.18*)	**0.90 (*0.18*)**	**0.76 (*0.31*)**	**0.70 (*0.33*)**	0.46 (*0.19*)	**0.66 (*0.22*)**	**Weak**
10^−3^	0.03 (*0.02*)	0.09 (*0.13*)	0.00 (*0.06*)	0.01 (*0.03*)	0.11 (*0.23*)	0.14 (*0.27*)	Moderate
10^−4^	−0.03 (*0.09*)	0.08 (*0.13*)	0.05 (*0.09*)	−0.07 (*0.05*)	0.11 (*0.22*)	0.13 (*0.20*)	Moderate
10^−5^	0.00 (*0.05*)	0.07 (*0.19*)	0.02 (*0.03*)	−0.08 (*0.01*)	0.11 (*0.26*)	0.09 (*0.19*)	High
10^−6^	0.04 (*0.10*)	0.12 (*0.14*)	0.04 (*0.03*)	−0.01 (*0.01*)	0.11 (*0.24*)	0.10 (*0.12*)	High
Average **BPc = 0.59 (*0.03*)**							
**(D) McF**	**CTR (+)**	**TIM**	**FEP**	**CAZ**	**IPM**	**MEM**	
10^−1^	**0.80 (*0.26*)**	**0.98 (*0.04*)**	**0.93 (*0.13*)**	**0.87 (*0.23*)**	1.00 (*0.00*)	**0.95 (*0.09*)**	**No**
10^−2^	0.15 (*0.22*)	0.24 (*0.07*)	0.19 (*0.17*)	0.09 (*0.01*)	**0.92 (*0.07*)**	0.14 (*0.02*)	**Weak**
10^−3^	0.03 (*0.08*)	0.26 (*0.21*)	0.19 (*0.28*)	0.18 (*0.24*)	0.16 (*0.09*)	0.03 (*0.07*)	Moderate
10^−4^	0.02 (*0.06*)	0.02 (*0.05*)	−0.03 (*0.03*)	−0.01 (*0.05*)	0.03 (*0.09*)	0.00 (*0.07*)	Moderate
10^−5^	−0.01 (*0.08*)	−0.01 (*0.01*)	−0.03 (*0.02*)	−0.04 (*0.02*)	0.01 (*0.08*)	−0.01 (*0.01*)	High
10^−6^	0.02 (*0.05*)	0.04 (*0.05*)	−0.01 (*0.05*)	−0.03 (*0.03*)	0.05 (*0.09*)	−0.01 (*0.04*)	High
Average **BPc = 0.58 (*0.02*)**							

Values reported in the table refer to the average Biofilm-forming Potential (BP) measured for each strain in culture with 0.1xMIC of β-lactams in triplicate, followed by its corresponding standard deviation (*SD*). This standardized value, ranging from 0 (no biofilm) to 1 (full biofilm) and defined by the formula BP = [1 − (BFI sample/average BFIs of negative control)], depicts the biofilm production by bacteria. Biofilm formations were obtained after 5 h of incubation at 37 °C. For each condition, the biofilm strength classification corresponded with the first BP value higher than the cut-off one of the experiments (BPc). Corresponding categorization included the no-, weak-, moderate- and high-biofilm producer categories. Abbreviations of antibiotics are TIM for ticarcillin-clavulanate, FEP for cefepime, CAZ for ceftazidime, IPM for imipenem, and MEM for meropenem.

**Table 2:** Bacteria/cluster size and number of microbial components associated with their corresponding variation rates observed for each strain +/− antibiotics in epifluorescence experiments.

**Strains**	**Cell/Cluster Size (µm)**	**Variation Rate (%)**				
	**CTR(+)**	**TIM**	**FEP**	**CAZ**	**IPM**	**MEM**
#6	**0.9**	+244% ***	+175% *	+193% ***	+43% ^ns^	+213% ****
#20	**49 ●**	+180% ●^ns^	+66% ●^ns^	−15% ●^ns^	+84% ●^ns^	−22% ●^ns^
#22	**1.2**	+299% ****	+329% ****	+389% ****	+113% ^ns^	+474% ****
#31	**1.1**	+78% ****	+58% ***	+47% **	−29% ^ns^	+35% ^ns^
**Strains**	**Cell/Cluster Number**	**Variation Rate (%)**				
	**CTR(+)**	**TIM**	**FEP**	**CAZ**	**IPM**	**MEM**
#6	**351**	−60% *	−54% *	−77% **	+82% **	−31% ^ns^
#20	**56 ●**	−10% ●^ns^	−2% ●^ns^	+7% ●^ns^	+14% ●^ns^	+89% ●^ns^
#22	**204**	−20% ^ns^	−73% *	−46% ^ns^	+203% ****	−37% ^ns^
#31	**567**	−76%*	−57% ^ns^	−38% ^ns^	+107% **	−66% ^ns^

ImageJ software allowed for the size calculation of each spotted fluorescent object by the conversion of their corresponding pixel number in µm. It allowed for the length evaluation of individual bacterial cells but when a strain spontaneously adheres, the formation of microbial micro-colonies could be observed and isolated cells were not easily detectable. In that case, the shape of overall clusters was estimated (values notified by the sign ● in the table). The image processing software also enabled the quantification of delimited objects visible on each acquired microscopic field. Either the number of bacteria or the number of corresponding clusters was documented. Data included in the table are average values arisen from the analysis of nine observation fields (fluorescent microscopy experiments were performed in triplicates and three pictures were captured for each condition). Statistical analyses were performed through one-way ANOVA and Dunnett’s methods for the comparison of each antimicrobial condition with their corresponding control strain (*, **, ***, **** adjusted *p*-values).

**Table 3:** β-lactam MICs and sub-inhibitory concentrations of clinical *P. aeruginosa* strains.

	(µg/mL)	TIM	FEP	CAZ	IPM	MEM
Strain #6	MIC	48	24	16	3	0.75
	**0.1 × MIC**	**4.8**	**2.4**	**1.6**	**0.3**	**0.075**
Strain #20	MIC	1	12	0.75	1.5	0.064
	**0.1 × MIC**	**0.1**	**1.2**	**0.075**	**0.15**	**0.0064**
Strain #22	MIC	256	96	128	16	32
	**0.1 × MIC**	**25.6**	**9.6**	**12.8**	**1.6**	**3.2**
Strain #31	MIC	24	1.5	1.5	3	1
	**0.1 × MIC**	**2.4**	**0.15**	**0.15**	**0.3**	**0.1**

Antibiotic MICs were initially determined by gradient diffusion strips.

## References

[1] Faure E., Kwong K., Nguyen D. (2018). *Pseudomonas aeruginosa* in Chronic Lung Infections: How to Adapt within the Host?. Front. Immunol..

[2] Nathwani D., Raman G., Sulham K., Gavaghan M., Menon V. (2014). Clinical and economic consequences of hospital-acquired resistant and multidrug-resistant *Pseudomonas aeruginosa* infections: A systematic review and meta-analysis. Antimicrob. Resist. Infect. Control.

[3] Chatterjee M., Anju C.P., Biswas L., Anil Kumar V., Gopi Mohan C., Biswas R. (2016). Antibiotic resistance in *Pseudomonas aeruginosa* and alternative therapeutic options. Int. J. Med. Microbiol..

[4] Hall C.W., Mah T.-F. (2017). Molecular mechanisms of biofilm-based antibiotic resistance and tolerance in pathogenic bacteria. FEMS Microbiol. Rev..

[5] Costerton J.W., Stewart P.S., Greenberg E.P. (1999). Bacterial biofilms: A common cause of persistent infections. Science.

[6] Venkatesan N., Perumal G., Doble M. (2015). Bacterial resistance in biofilm-associated bacteria. Future Microbiol..

[7] Aminov R.I. (2013). Biotic acts of antibiotics. Front. Microbiol..

[8] Linares J.F., Gustafsson I., Baquero F., Martinez J.L. (2006). Antibiotics as intermicrobial signaling agents instead of weapons. Proc. Natl. Acad. Sci. USA.

[9] Hathroubi S., Mekni M.A., Domenico P., Nguyen D., Jacques M. (2017). Biofilms: Microbial Shelters Against Antibiotics. Microb. Drug Resist..

[10] Kaplan J.B. (2011). Antibiotic-induced biofilm formation. Int. J. Artif. Organs.

[11] Lund-Palau H., Turnbull A.R., Bush A., Bardin E., Cameron L., Soren O., Wierre-Gore N., Alton E.W., Bundy J.G., Connett G. (2016). *Pseudomonas aeruginosa* infection in cystic fibrosis: Pathophysiological mechanisms and therapeutic approaches. Expert Rev. Respir. Med..

[12] Olivares E., Badel-Berchoux S., Provot C., Jaulhac B., Prévost G., Bernardi T., Jehl F. (2016). The BioFilm Ring Test: A Rapid Method for Routine Analysis of *Pseudomonas aeruginosa* Biofilm Formation Kinetics. J. Clin. Microbiol..

[13] Olivares E., Badel-Berchoux S., Provot C., Jaulhac B., Prévost G., Bernardi T., Jehl F. (2017). Tobramycin and Amikacin Delay Adhesion and Microcolony Formation in *Pseudomonas aeruginosa* Cystic Fibrosis Isolates. Front. Microbiol..

[14] Di Domenico E.G., Toma L., Provot C., Ascenzioni F., Sperduti I., Prignano G., Gallo M.T., Pimpinelli F., Bordignon V., Bernardi T. (2016). Development of an *in vitro* Assay, Based on the BioFilm Ring Test®, for Rapid Profiling of Biofilm-Growing Bacteria. Front. Microbiol..

[15] EUCAST: New S, I and R Definitions. https://www.eucast.org/newsiandr/.

[16] Kaplan J.B., Izano E.A., Gopal P., Karwacki M.T., Kim S., Bose J.L., Bayles K.W., Horswill A.R. (2012). Low Levels of β-Lactam Antibiotics Induce Extracellular DNA Release and Biofilm Formation in *Staphylococcus aureus*. mBio.

[17] Nucleo E., Steffanoni L., Fugazza G., Migliavacca R., Giacobone E., Navarra A., Pagani L., Landini P. (2009). Growth in glucose-based medium and exposure to subinhibitory concentrations of imipenem induce biofilm formation in a multidrug-resistant clinical isolate of *Acinetobacter baumannii*. BMC Microbiol..

[18] Macià M.D., Rojo-Molinero E., Oliver A. (2014). Antimicrobial susceptibility testing in biofilm growing bacteria. Clin. Microbiol. Infect..

[19] Eladawy M., El-Mowafy M., El-Sokkary M.M.A., Barwa R. (2020). Effects of Lysozyme, Proteinase K, and Cephalosporins on Biofilm Formation by Clinical Isolates of *Pseudomonas aeruginosa*. Interdiscip. Perspect. Infect. Dis..

[20] Perez L.R.R., Costa M.C.N., Freitas A.L.P.D., Barth A.L. (2011). Evaluation of biofilm production by *Pseudomonas aeruginosa* isolates recovered from cystic fibrosis and non-cystic fibrosis patients. Braz. J. Microbiol..

[21] Penesyan A., Paulsen I.T., Gillings M.R., Kjelleberg S., Manefield M.J. (2020). Secondary Effects of Antibiotics on Microbial Biofilms. Front. Microbiol..

[22] Sousa A.M., Pereira M.O. (2014). *Pseudomonas aeruginosa* diversification during infection development in cystic fibrosis lungs—A review. Pathogens.

[23] Oliver A., Cantón R., Campo P., Baquero F., Blázquez J. (2000). High Frequency of Hypermutable *Pseudomonas aeruginosa* in Cystic Fibrosis Lung Infection. Science.

[24] Song T., Duperthuy M., Wai S.N. (2016). Sub-Optimal Treatment of Bacterial Biofilms. Antibiotics.

[25] Horii T., Kobayashi M., Sato K., Ichiyama S., Ohta M. (1998). An *in vitro* study of carbapenem-induced morphological changes and endotoxin release in clinical isolates of gram-negative bacilli. J. Antimicrob. Chemother..

[26] Jackson J.J., Kropp H. (1996). Differences in mode of action of β-lactam antibiotics influence morphology, LPS release and in vivo antibiotic efficacy. J. Endotoxin Res..

[27] Buijs J., Dofferhoff A.S.M., Mouton J.W., Wagenvoort J.H.T., van der Meer J.W.M. (2008). Concentration-dependency of β-lactam-induced filament formation in Gram-negative bacteria. Clin. Microbiol. Infect..

[28] Pucci M.J., Boice-Sowek J., Kessler R.E., Dougherty T.J. (1991). Comparison of cefepime, cefpirome, and cefaclidine binding affinities for penicillin-binding proteins in *Escherichia coli* K-12 and *Pseudomonas aeruginosa* SC8329. Antimicrob. Agents Chemother..

[29] Elliott T.S., Greenwood D. (1983). The response of *Pseudomonas aeruginosa* to azlocillin, ticarcillin and cefsulodin. J. Med. Microbiol..

[30] Oura H., Tashiro Y., Toyofuku M., Ueda K., Kiyokawa T., Ito S., Takahashi Y., Lee S., Nojiri H., Nakajima-Kambe T. (2015). Inhibition of *Pseudomonas aeruginosa* Swarming Motility by 1-Naphthol and Other Bicyclic Compounds Bearing Hydroxyl Groups. Appl. Environ. Microbiol..

[31] Van Teeseling M.C.F., de Pedro M.A., Cava F. (2017). Determinants of Bacterial Morphology: From Fundamentals to Possibilities for Antimicrobial Targeting. Front. Microbiol..

[32] Rasamiravaka T., Labtani Q., Duez P., El Jaziri M. (2015). The Formation of Biofilms by *Pseudomonas aeruginosa*: A Review of the Natural and Synthetic Compounds Interfering with Control Mechanisms. Biomed. Res. Int..

[33] Vijayakumar S., Rajenderan S., Laishram S., Anandan S., Balaji V., Biswas I. (2016). Biofilm Formation and Motility Depend on the Nature of the *Acinetobacter baumannii* Clinical Isolates. Front. Public Health.

[34] Hoffman L.R., D’Argenio D.A., MacCoss M.J., Zhang Z., Jones R.A., Miller S.I. (2005). Aminoglycoside antibiotics induce bacterial biofilm formation. Nature.

[35] Ratcliff W.C., Denison R.F. (2011). Alternative Actions for Antibiotics. Science.

[36] Cornforth D.M., Foster K.R. (2013). Competition sensing: The social side of bacterial stress responses. Nat. Rev. Micro..

[37] Elliott D., Burns J.L., Hoffman L.R. (2010). Exploratory Study of the Prevalence and Clinical Significance of Tobramycin-Mediated Biofilm Induction in *Pseudomonas aeruginosa* Isolates from Cystic Fibrosis Patients. Antimicrob. Agents Chemother..

[38] Odenholt I. (2001). Pharmacodynamic effects of subinhibitory antibiotic concentrations. Int. J. Antimicrob. Agents.

[39] Hurley M.N., Ariff A.H.A., Bertenshaw C., Bhatt J., Smyth A.R. (2012). Results of antibiotic susceptibility testing do not influence clinical outcome in children with cystic fibrosis. J. Cyst. Fibros..

